# Cognitive impairment after lacunar stroke: systematic review and meta-analysis of incidence, prevalence and comparison with other stroke subtypes

**DOI:** 10.1136/jnnp-2012-303645

**Published:** 2013-03-01

**Authors:** Stephen David James Makin, Sarah Turpin, Martin S Dennis, Joanna M Wardlaw

**Affiliations:** 1 Division of Clinical Neurosciences, University of Edinburgh, Edinburgh, UK; 2 Department of Geriatric Medicine, Liberton Hospital, NHS Lothian, Edinburgh, UK; 3 Division of Clinical Neuroimaging Sciences, University of Edinburgh, Edinburgh, UK

**Keywords:** Stroke, Cognition, Dementia

## Abstract

**Background:**

Cognitive impairment and dementia are common after stroke. It is unclear if risk differs between ischaemic stroke subtypes. Lacunar strokes might be less likely to affect cognition than more severe, larger cortical strokes, except that lacunar strokes are associated with cerebral small vessel disease (SVD), which is the commonest vascular cause of dementia.

**Methods:**

We searched MEDLINE and PsychINFO for studies of mild cognitive impairment (MCI) or dementia after lacunar or cortical ischaemic stroke. We calculated the OR for cognitive impairment/dementia in lacunar versus non-lacunar stroke, and their incidence and prevalence in lacunar stroke as a pooled proportion.

**Findings:**

We identified 24 relevant studies of 7575 patients, including 2860 with lacunar stroke; 24% had MCI or dementia post stroke. Similar proportions of patients with lacunar and non-lacunar stroke (16 studies, n=6478) had MCI or dementia up to 4 years after stroke (OR 0.72 (95% CI 0.43 to 1.20)). The prevalence of dementia after lacunar stroke (six studies, n=1421) was 20% (95% CI 9 to 33) and the incidence of MCI or dementia (four studies, n=275) was 37% (95% CI 23 to 53). Data were limited by short follow-up, subtype classification methods and confounding.

**Interpretation:**

Cognitive impairment appears to be common after lacunar strokes despite their small size, suggesting that associated SVD may increase their impact. New prospective studies are required with accurate stroke subtyping to assess long term outcomes while accounting for confounders.

## Introduction

Dementia is common soon after stroke^1^ but we know little of the mechanisms or whether the risk varies with stroke subtype. Stroke risk factors, amounts and regions of the brain affected, and suggested stroke mechanisms all vary with stroke subtype, and could influence the risk of cognitive impairment after stroke. For example, as cortical ischaemic strokes often affect a large area of brain, they may carry a higher risk of cognitive impairment than the smaller, less neurologically severe, lacunar strokes. Alternatively, lacunar strokes may carry a higher risk of cognitive impairment than would be expected on the basis of the lacunar infarct alone as they are part of the spectrum of cerebral small vessel disease (SVD). SVD, which affects the brain diffusely and is the commonest vascular cause of cognitive impairment,^2^ could be unmasked by a new lacunar stroke. A recent systematic review^3^ included studies of patients after lacunar stroke who had undergone detailed neuropsychological assessments but did not provide information on whether lacunar stroke patients are at greater risk than other stroke subtypes, or on potential confounding factors.

We sought to establish the incidence and prevalence of cognitive impairment and dementia after lacunar stroke, in the short and long term, and its magnitude in comparison with cortical stroke, by systematically reviewing the available literature. We also aimed to evaluate study quality and whether they had accounted for confounding factors that may affect performance on cognitive tests such as depression, lower premorbid IQ and pre-existing dementia or SVD.

## Methods

### Search methods

Following guidance from experienced librarians from the Cochrane Stroke Group, we searched MEDLINE (1991 to present) and PsychINFO (1991 to present) with the terms (a) ‘cognition’ ‘dementia’ ‘vascular dementia’ or ‘multi-infarct dementia’ and (b) ‘stroke’ or ‘lacunar stroke’ (last searched 7 June 2012, using OVID SP V.03.07.00.119, details in the online supplementary information). We checked references in review articles and hand searched the previous 5 years of *Stroke* and *Age and Aging*. We complied with the meta-analysis of observational systematic reviews (MOOSE)^4^ group guidelines.

### Study criteria

In our primary analysis we included studies that compared cognition in patients with lacunar stroke with those with cortical stroke. Our secondary analysis to determine the incidence and prevalence of mild cognitive impairment (MCI) and dementia analysed all studies that measured cognition in patients with lacunar stroke.

We included studies that assessed MCI and dementia in adult humans with symptomatic lacunar stroke (defined below). We excluded studies of asymptomatic patients with lesions on imaging and autopsy studies.

In order to establish the proportion of lacunar stroke patients with MCI or dementia, we included studies that tested an unselected group of patients. We excluded studies that tested a very selected group of patients—for example, a study of only patients with a particular radiological finding such as a recent lacunar infarct in the thalamus.

We included English and non-English language studies. We excluded studies that were published only in abstract and those which had tested a group of stroke patients, including some with lacunar stroke, but not presented the results according to stroke subtype.

### Definitions of lacunar stroke, dementia and MCI

We set the reference standard for stroke subtyping as a non-risk factor dependent clinically based approach (such as the Oxfordshire Community Stroke Project, OSCP)^5^ accompanied by MRI, including diffusion weighted imaging (DWI), to identify the recent acute ischaemic lesion. We also accepted studies which made the diagnosis of stroke subtype in other ways, such as clinical syndrome plus CT scanning instead of MR with DWI. We defined a lacunar stroke as one diagnosed as ‘lacunar’ on clinical grounds (a typical lacunar syndrome, or using methods devised by Bamford *et al*^5^ in the OCSP or similar method) with or without imaging verification, or as a clinically evident stroke classed as ‘small vessel’ by the risk factor based classifications such as the Trial of ORG 10172 in Acute Stroke Treatment (TOAST) classification.^6^ We defined non-lacunar stroke as a sudden onset of neurological symptoms classed as ‘cortical stroke’ clinically with or without imaging verification, or a stroke classed as ‘large artery atherosclerosis’, ‘cardioembolism’, ‘cryptogenic’ or ‘other’ on TOAST, or other risk factor based classifications.^6^ We noted the definition used in each study so as to perform sensitivity analyses.

We set the reference standard for cognitive impairment as a full neuropsychological assessment covering all cognitive domains, blinded to stroke subtype. We defined dementia as impairment of cognitive function which interfered with everyday activities, and we defined MCI as impairment of cognitive function not severe enough to interfere with everyday life.^7^ We used the term ‘cognitive impairment’ to refer to any impairment of cognitive function whether MCI or dementia.

We included studies which had made the diagnosis of cognitive impairment or dementia in other ways, such as the Mini-Mental State Examination (MMSE). We used the criteria for cognitive impairment, MCI or dementia as applied in individual studies.

### Data extraction

We used a pretested form to extract available data. We extracted the following data about patient recruitment and assessment methods: inclusion and exclusion criteria (specifically whether the study included patients with prior stroke, prior cognitive impairment or aphasia, and if so whether adapted tests were used); how stroke subtypes were defined and if there were any differences between the subtypes; whether information was sought from relatives about signs of cognitive decline prior to stroke; and the details of assessments, including whether depression or premorbid IQ were measured and accounted for, what proportion of patients were able to complete cognitive testing and whether the cognitive assessors were blinded to the clinical information. We extracted the details about the study population: ages and risk factors; number of patients with lacunar or non-lacunar stroke and how many were recorded as having dementia and/or MCI; if impairments occurred in any particular cognitive domain; and any calculated odds or HRs and whether these were adjusted for risk factors, including SVD on imaging, depression and premorbid IQ.

If a study had published more than one paper, we ensured that each patient only contributed once towards the present analysis. The studies were reviewed and the data extracted by two of the authors (SDJM and ST) with advice from another author (JMW). We did not contact the authors to obtain additional information.

### Statistical analysis

Firstly we investigated whether patients with lacunar stroke were more likely to have cognitive impairment than patients with non-lacunar stroke by calculating the pooled OR and 95% CI of cognitive impairment in patients with lacunar compared with cortical stroke (the OR would be >1.0 if cognitive impairment were more common in lacunar stroke).

We examined the impact of potential confounders by recording available adjusted outcome measures and by analysis of the following prespecified subgroups: community versus hospital based studies; timing of cognitive assessment after stroke (early=under 1 month, mid=1 month to 1 year and later than 1 year); whether or not patients with previous stroke and/or dementia had been excluded; stroke subtyping method used; cognitive test used; whether depression or premorbid IQ were accounted for; whether patients had MRI or CT imaging to aid diagnosis of subtype; and the proportion of patients who were cognitively assessed. Secondly, we aimed to calculate the incidence and prevalence of cognitive impairment and dementia after lacunar stroke. We calculated the OR and 95% CI using a weighted Mantel–Haenszel summary OR in Review Manager V.5.1. We calculated the incidence and prevalence of cognitive impairment and dementia in patients with lacunar stroke as a pooled proportion and 95% CI, using a DerSimonian Laird random effects model in StatsDirect V.2.7.8. We assessed for heterogeneity by calculating the I^2^ statistic for each meta-analysis and publication bias using a funnel plot. We used a random effects model due to potential heterogeneity in the underlying methodology. No ethics approval was necessary in order to conduct this literature review as it was all literature based.

## Results

**Figure 1:**
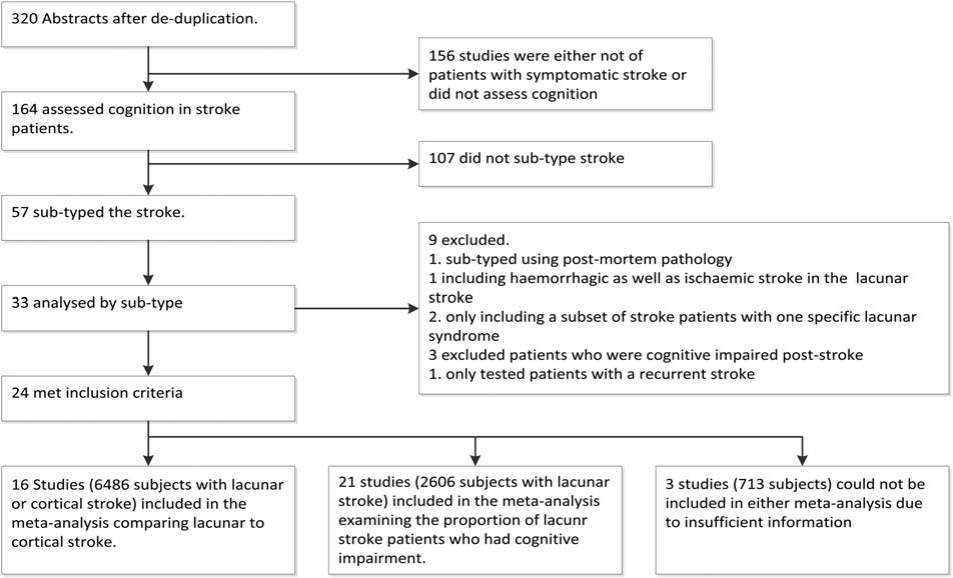
Flow diagram of search strategy and results.

Of 320 potentially suitable papers, 164 assessed cognition in stroke patients but only 57 assessed both cognitive function and stroke subtype; of these, 33 presented the results by subtype but nine did not meet other prespecified criteria (see details in the online supplementary table), leaving 24 that met our inclusion criteria (figure 1). The 24 studies included 7575 subjects with ischaemic stroke (table 1).

**Table 1:** Characteristics of included studies

Reference	Setting	Total patients consented/total tested	Timing of tests post-stroke	Test done	Subtyping	Primary outcome	Results
Nys 2007^8^ ^9^	Hospital The Netherlands	190/168*	3 weeks	NP†	Imaging	MCI‡	29/64 (45%)§ Lacunar 47/63 (75%)§ Non-lacunar
Sachdev *et al*^10^	Hospital Australia	210/170*	3–6 months	NP	Risk factor	Dementia or MCI	24/46 (52%) Lacunar 74/120 (62%) Non-lacunar
De Koning *et al*^11^	Hospital The Netherlands	130/121*	3–9 months	NP	Imaging	Dementia	4/20 (20%) Lacunar¶ 17/38 (45%) Non-lacunar¶
Censori *et al*^12^	Hospital Italy	121/110**	3–4 Months	NP and MMSE††	Clinical	Dementia	2/21 (10%) Lacunar 13/83 (16%)Non-lacunar
Tatemichi *et al*^13^	Hospital USA	927/726	2 years	Clinical impression	Risk factor	Dementia	25/227 (11%) Lacunar 91/499 (18%) Non-lacunar
Bejot *et al*^14^	Community France	3948/3201*	1 month	NP	Risk factor	Dementia	333/887 (38%) Lacunar 289/1960 (15%) Non-lacunar
Lin *et al*^15^	Hospital Taiwan	353/283	3 months	NP	Risk factor	Dementia	13/136 (10%) Lacunar 13/147 (9%) Non-lacunar
Patel *et al*^16^ ^17^	Community UK	1454/654*	3 months	MMSE	Clinical	MMSE<24	72/218 (33%) Lacunar 124/297 (42%) Non-lacunar
Cordoliani-Mackowiak *et al*^18^	Hospital France	132/88‡‡	Up to 3 years	NP	Risk factor	Dementia	8/31 (26%) Lacunar 24/101 (24%) Non-lacunar
Rasquin *et al*^19–22^	Hospital The Netherlands	176/144	1 years	NP	Imaging	MCI or Dementia	40/57 (70%) Lacunar 59/87 (68%) Non-lacunar^§§^
Pohjasvaara *et al*^23^	Hospital Finland	451/337	3 months	NP	Risk factor	Dementia	5/21 (24%) Lacunar 102/316 (32%) Non-lacunar
Tang *et al*^24^	Hospital China	484/280*	3 months	MMSE	Imaging	Dementia	33/166 (20%) Lacunar 18/90 (20%) Non-lacunar
Madureira *et al*^25^	Hospital Portugal	180/165	3 months	NP	Imaging	Dementia	6/139 (4.3%) Lacunar¶¶ 5/41 (12%) Non-lacunar¶¶
Klimkowicz-Mrowiec *et al*^26^	Hospital Poland	173/145‡‡*	3 months	NP and MMSE	Clnical	Dementia	2/24 (8%) Lacunar 35/149 (23%) Non-lacunar
Dong *et al*^27^	Hospital Singapore	300/239*	3–6 months	NP, MMSE, and MoCA§§	Risk factor	Moderate MCI	21/106 (20%) Lacunar 31/97 (33%) Non-lacunar
Tatemichi *et al*^28^	Hospital USA	Not stated/227	3 months	NP	Clinical	MCI	21/59 (36%) Lacunar 59/168 (35%) Non-lacunar
Tay *et al*^29^	Hospital Singapore	216/169	9 days	MMSE	Clinical	NA	Mean MMSE Non-lacunar (anterior circulation)18.2, Lacunar 22.9
Mok *et al*^30^	Hospital China	86/75	3 months***	MMSE and clinical dementia rating scale	Imaging	Dementia	10/75 (13%) Mean MMSE: 24.8 lacunar, 27.7 healthy controls
Samuelsson *et al*^31^	Hospital Sweden	100/81	2 years	MMSE and NP if impaired.	Clinical	Dementia	8/81 (10%)
Anderson *et al*^32^	Community Australia	Not stated/30	1 year	NP	Clinical	MCI	2/30 (7%)
Loeb *et al*^33^	Hospital Italy	Not stated/108	Up to 4 years	MMSE	Clinical	Dementia	25/108 (23%)
Fure *et al*^34^	Hospital Norway	71/64‡‡	2–7 days	NP	Risk factor	MCI	41/71 (58%)
Barker-Collo *et al*^35^	Community New Zealand	357/336	5 years	NP	Clinical	Cognition test results.	No difference in mean test scores
Appelros *et al*^36^	Community Sweden	253/232*	1 year	MMSE	Risk factor	Mean MMSE scores	Mean MMSE Non-lacunar 25.6, Lacunar 26.6

*Includes subjects with haemorrhagic stroke/transient ischaemic attack.

†Full neuropsychological (NP) testing.

‡Mild cognitive impairment.

§14 haemorrhagic strokes included in these figures.

¶Subtype reported only in patients who had a visible lesion on CT.

**Six subjects with specific cognitive impairment excluded.

††Mini-Mental State Examination.

‡‡Data reported on subjects who could not have full tests.

§§Calculated from reported OR.

¶¶Calculated from χ^2^ statistic.

***The 61 non-demented patients were followed up 2–3 years later; no information on outcome of those who did have dementia at 3 months.^37^

§§MoCA, Montreal Cognitive Assessment.

Nineteen studies compared lacunar with non-lacunar stroke^9–29^ ^35^ ^36^ (79% of studies, 95% of subjects), three studies^30^ ^32^ ^33^ compared lacunar stroke with healthy volunteers and two had no control group. Median study size was 170 patients (range 30–3201) and mean age of the patients was 73 years (we were not able to calculate median age).

Assessment of cognition, potential confounders and reporting of results varied. Seventeen studies^9–17^ ^19–25^ ^27^ ^29^ ^30^ ^35^ ^36^ reported the number of subjects in whom testing was attempted (9154 patients) and the number that completed the tests (7401 patients); eight^9–12^ ^14^ ^16^ ^17^ ^24^ ^27^ ^36^ studies reported the combined number of attempted and successful tests in patients with ischaemic and haemorrhagic stroke, but not the values for ischaemic stroke alone; and seven studies^18^ ^25^ ^26^ ^29^ ^31^ ^34^ ^35^ reported cognitive outcomes for 155 patients who only completed some of the assessments. Two studies 12 30 excluded patients with depression (8% of studies, 2% of patients); one study^10^ measured premorbid IQ using the National Adult Reading Test but did not account for it in the analysis (4% of studies, 3% of patients); and nine studies^9–11^ ^15^ ^18^ ^24^ ^26^ ^27^ ^30^ interviewed relatives about signs of cognitive decline prior to the index stroke (38% of studies, 19% of patients). Three studies^9^ ^24^ ^25^ stated that the assessors were blinded to the stroke subtype (13% of studies, 7% of patients) but the rest did not mention blinding. Some information on background SVD was obtained in 10 studies but the varied methods and reporting precluded its further use: one study (n=81) measured white matter lesion (WML) volume on MRI in all patients;^31^ another (n=170) measured WML volume in 133/170 patients who had MRI^10^; and eight studies^11^ ^12^ ^18^ ^21^ ^24^ ^26^ ^30^ ^33^ scored leukoaraiosis on CT scans of all patients (33% of studies, 16% of patients).

Stroke subtyping methods and use of imaging in subtype diagnosis varied. All studies required a clinical diagnosis of a stroke alongside imaging to exclude haemorrhage. Two studies^23^ ^31^ performed MRI on all patients but did not state whether or not this included DWI (8% of studies, 5% of patients), a further 11^11–13^ ^18^ ^24^ ^26^ ^28^ ^30^ ^33^ ^34^ ^36^ performed CT scanning on all patients (46% of studies, 28% of subjects). The remaining studies either performed a mixture of CT and MRI scans or did not give imaging details. Nine studies^12^ ^16^ ^26^ ^29^ ^31^ ^32^ ^28^ ^33^ ^35^ used a risk factor free clinical classification (eg, OCSP^5^) to categorise subjects as ‘lacunar’ or ‘non-lacunar’ stroke (38% of studies, 23% of patients). Nine studies^10^ ^13–15^ ^18^ ^23^ ^27^ ^34^ ^36^ used the risk factor based TOAST classification (38% of studies, 66% of patients). Six studies^9^ ^11^ ^20^ ^24^ ^25^ ^30^ used imaging features alone to subtype the clinically apparent stroke (25% of studies, 11% of patients).

The extent of cognitive testing varied. Sixteen studies^9–12^ ^14^ ^15^ ^18^ ^21^ ^23^ ^25–28^ ^32^ ^34^ ^35^ performed detailed cognitive testing of all cognitive domains (67% of studies, 72% of patients). Six studies^16^ ^24^ ^29^ ^31^ ^33^ ^36^ used only the MMSE (25% of studies, 18% of patients). One^11^ (n=58) used the Rotterdam Cambridge Cognitive Assessment (R-CAMCOG) while another^30^ (n=75) used the Alzheimer's Disease Assessment Scale cognitive subscale (ADAS-COG) and MMSE. One study^13^ (n=726) based cognitive assessment on the assessing clinician's impression of whether the patient was demented. Two studies^10^ ^20^ recorded the number of subjects with MCI but no dementia. One study^35^ (n=336) reported test results across OCSP subtypes: lacunar patients performed worse on the ‘matrix reasoning’ test of visuoperceptual functioning and anterior (not posterior) circulation cortical stroke patients performed worse on the Stroop test of executive function; neither difference was seen on other tests of the same function.

The timing of the cognitive assessment varied. Four studies^9^ ^14^ ^29^ ^34^ assessed cognition in the first month after stroke (17% of studies, 42% of patients). Twelve studies^10–12^ ^15^ ^16^ ^23–28^ ^30^ assessed cognition between 3 months and 1 year (50% studies, 34% patients), and eight^13^ ^18^ ^20^ ^31–33^ ^35^ ^36^ assessed cognition between 1 and 4 years after stroke (33% studies, 24% patients).

**Figure 2:**
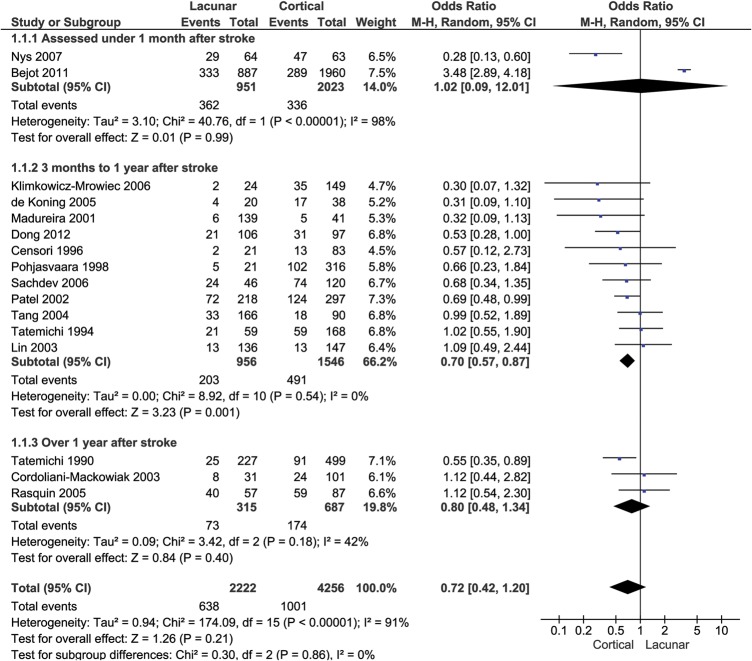
Odds of cognitive impairment in lacunar stroke versus cortical stroke.

**Figure 3:**
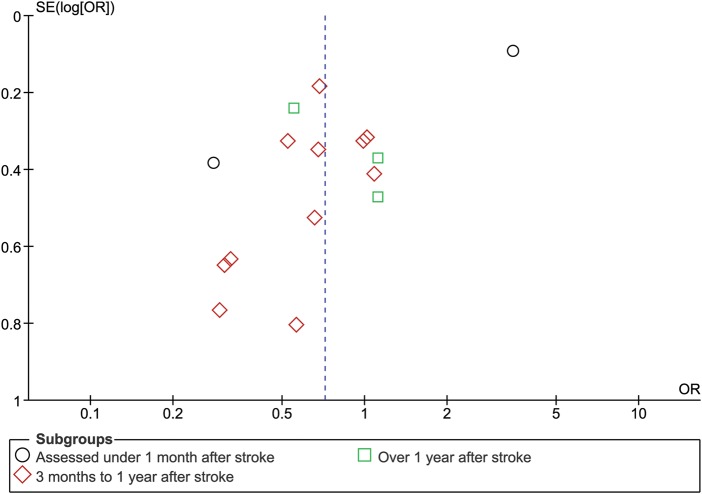
Funnel plot demonstrating that the heterogeneity was not due to publication bias. This figure is only reproduced in colour in the online version.

We compared the risk of cognitive impairment in patients with lacunar stroke with those with non-lacunar stroke (figure 2). As we were not able to analyse studies that only presented mean cognitive test scores nor those without a control group, only 16/24 studies^9–16^ ^18^ ^22–28^ of 6478 subjects contributed to this analysis. The proportion of patients with cognitive impairment—either MCI or dementia—did not differ between patients with lacunar and non-lacunar stroke: 638/2222 (29%) subjects with lacunar stroke compared with 1001/4256 (24%) subjects with non-lacunar stroke (OR 0.72; 95% CI 0.43 to 1.20). The substantial heterogeneity between the studies (I^2^ 91%; p=0.00001) was not apparently due to publication bias (figure 3) or to differences in the time elapsed between the index stroke and assessment of cognition (figure 2).

**Figure 4:**
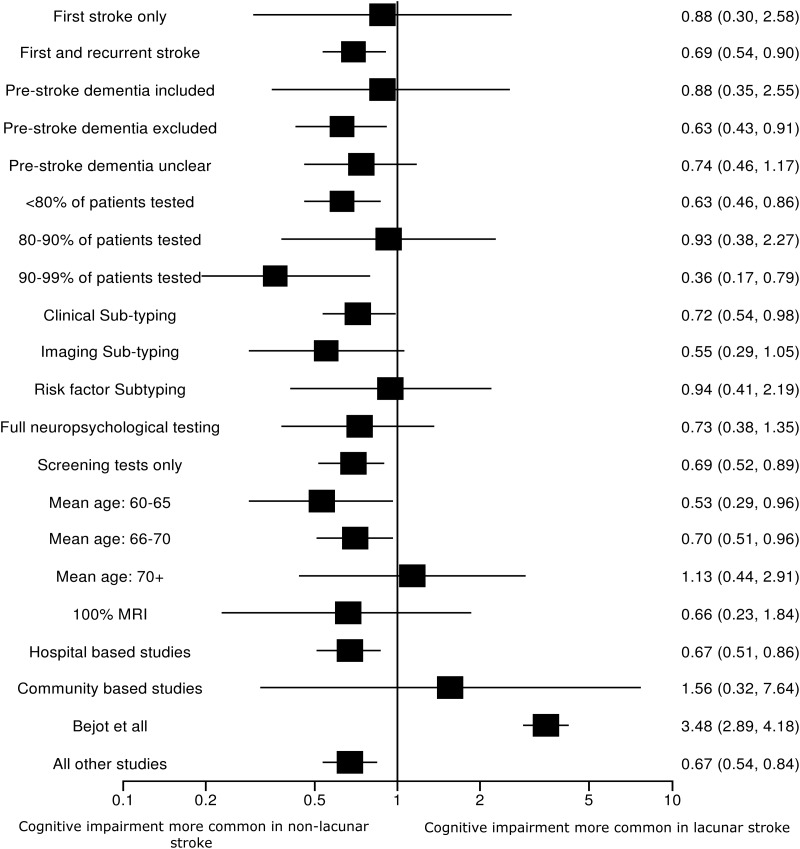
Sensitivity analysis: OR of cognitive impairment in lacunar against cortical stroke for studies with particular characteristics.

We performed a sensitivity analyses (figure 4) by sequentially excluding studies with particular characteristics and then repeating the analysis. Among hospital based studies, cognitive impairment was more common in non-lacunar stroke (OR 0.67; 95% CI 0.51 to 0.86); in community based studies, cognitive impairment was more common in lacunar stroke (OR 1.56; 95% CI 0.32 to 7.64) but with considerable heterogeneity (I^2^ 98%; p<0.001). There was little difference in the summary OR between studies that only included first stroke (OR 0.88; 95% CI 0.3 to 2.58), first or recurrent stroke (OR 0.69; 95% CI 0.54 to 0.9), where subjects with prior dementia were excluded (OR 0.63; 95% CI 43 to 0.91) or included (OR 0.88; 95% CI 0.35 to 2.55), between studies that tested smaller or larger numbers of participants (OR 0.63; 95% CI 0.46 to 0.86), for studies testing <80% (OR 0.63; 95% CI 0.46 to 0.86) against studies testing >80% of patients (OR 0.72; 95% CI 0.33 to 1.57), or according to patient age, whether limited or full neuropsychological testing was performed, or by the proportion of patients who had MRI scanning. Additionally, there was little difference in the summary OR between studies that used clinical, imaging or risk factor based subtyping to diagnosis lacunar stroke (see online supplementary figure). We did not have enough information to assess the impact of SVD on imaging. No studies provided a multivariant analysis of the risk of cognition in lacunar stroke adjusted for imaging evidence of SVD, depression, age or other confounders.

Heterogeneity was due to one large study^14^ (n=2847) in which cognitive impairment was more common in lacunar stroke (OR 3.48, 95% CI 2.89 to 4.18). When the meta-analysis was repeated after omitting this study, the OR changed from 0.72 (95% CI 0.43 to 1.20) with significant heterogeneity (as above) to 0.67 (95% CI 0.54 to 0.84) without significant heterogeneity (I^2^ 26%, p=0.17).

For the second meta-analysis, we aimed to establish the incidence and prevalence of cognitive impairment and dementia after a lacunar stroke. We included data from all studies that had measured cognition in patients with a lacunar stroke (a total of 21 studies of 2606 lacunar strokes) whether or not there was a control group. We were not able to include those studies that reported only average cognitive test results and did not dichotomise patients into ‘impaired’ and ‘not impaired’. A pooled proportion of eight^9^ ^10^ ^20^ ^27^ ^28^ ^32–34^ studies of 541 patients found that 38% (95% CI 24 to 52, I^2^ 92%) had MCI or dementia at any stage after a first or recurrent lacunar stroke. Fifteen studies provided data on dementia at any time after lacunar stroke (16%, 95% CI 10 to 24, I^2^ 93.8%)^10–16^ ^18^ ^22–26^ ^30^ ^31^

We then calculated the incidence (table 2) of dementia or MCI from studies that excluded patients with these conditions prior to stroke. One small study^12^ (n=21) found that the incidence of dementia was 10% (2/21; 95% CI 2 to 30) after a first lacunar stroke and another study^10^ (n=46) found that the incidence of MCI was 34% (16/46; 95% CI 22 to 49). Six studies^10^ ^12^ ^15^ ^18^ ^25^ ^26^ (n=397) found that the incidence of dementia after a first or recurrent lacunar stroke was 12% (95% CI 6 to 18). No studies of first and recurrent lacunar stroke recorded the incidence of MCI alone.

**Table 2:** Studies assessing incidence and prevalence of dementia and mild cognitive impairment in patients with lacunar stroke

	No of studies	No of patients	Pooled risk or proportion (95% CI) (%)	I^2^ (95% CI) (%)
Incidence of dementia (only previously non-demented patients included)				
1st stroke	1	21	10 (2 to 27)	n/a
1st or recurrent stroke	6	397	12 (6 to 18)	66.4 (0 to 89)
Prevalence of dementia (where the authors specified that they included patients who had dementia prior to the stroke)				
1st stroke	2	987	21 (1 to 55)	n/a
1st or recurrent stroke	6	1421	20 (9 to 33)	95.6 (93.4 to 96.8)
Prevalence of dementia (including studies where the methods were unclear and the authors did not state whether they included patients with dementia)				
1st stroke	4	1262	20 (8 to 36)	95.9 (93 to 97)
1st or recurrent stroke	9	1777	18 (9.6 to 30)	95.7 (95 to 96.7)
Incidence of MCI or dementia (only previously cognitively intact patients included)				
1st stroke	1	64	45 (34 to 58)	n/a
1st or recurrent stroke	4	275	37 (23 to 53)	85.5 (54 to 93)
Prevalence of MCI or dementia (where the authors specifically included prior dementia and MCI)				
No studies				
Prevalence of MCI or dementia (including studies where the methods were unclear)				
1st stroke	2	120	34 (0 to 9)	n/a
1st or recurrent stroke	4	266	38 (13 to 66)	96 (93.3 to 97.3)

MCI, mild cognitive impairment.

We planned to calculate the prevalence of dementia or MCI from studies that had included patients who had these conditions prior to the index stroke but no studies did this for MCI. Six studies provided these data for patients with dementia, two^14^ ^31^ (n=987) for first stroke (pooled percentage 21%; 95% CI 10 to 55) and six^11^ ^13^ ^14^ ^23^ ^24^ ^31^ (n=1421) for first or recurrent lacunar stroke (pooled percentage 20%; 95% CI 9 to 33).

## Discussion

The available literature suggests that approximately 30% of patients will be cognitively impaired in the 4 years following a lacunar stroke, a similar proportion to non-lacunar stroke (23%). However, precision and reliability of the estimate were hampered by several factors. Thus despite SVD being the commonest vascular cause of cognitive impairment and lacunar stroke often affecting people in middle not just old age, data to calculate the true incidence or prevalence of cognitive impairment after lacunar stroke and associated risk factors in order to advise patients and plan services are lacking.

A recent review^3^ summarised domain specific cognitive impairment after lacunar stroke but did not provide information on the proportion of lacunar stroke patients who are cognitively impaired, nor on whether this differs from non-lacunar stroke, or on the outcome of patients who were not able to have detailed cognitive assessment, nor the effect of depression, premorbid IQ or imaging evidence of SVD. However, it did highlight that when impairment does occur, it is generalised rather than domain specific.

The limitations of the literature included the fact that many studies were small, few assessors were blinded to the stroke subtype, most studies did not account for important confounders, and the diagnostic methods and reporting varied considerably. Importantly, there were few data on cognition beyond 1 year after the index stroke, yet lacunar stroke commonly affects patients in their 50s and 60s with many years of life and employment ahead. One study of 108^33^ patients performed follow-up as late as 4 years, another^35^ assessed cognitive function up to 5 years but reported only the mean test scores, not the number of patients with MCI or dementia. No studies used the reference standard method to diagnose lacunar stroke and differentiate it from non-lacunar stroke (risk factor free clinical subtyping supported by MRI–DWI in the acute phase). We could not perform risk factor adjusted analysis due to lack of data. Potential confounding factors were poorly addressed: two studies accounted for depression,^12^ ^30^ only one^10^ measured premorbid IQ (although 10 assessed for signs of pre-stroke cognitive impairment) and data were inadequate to assess the effect of background white matter hyperintensities. Most studies were hospital based, but as lacunar stroke may be mild, not all cases are admitted to hospital. Our calculations of incidence and prevalence were hampered by many studies not reporting whether the included patients had cognitive impairment or dementia prior to the stroke.

The estimate of cognitive impairment after lacunar versus non-lacunar stroke was strongly influenced by one study^14^ in which dementia was much more common in lacunar than in non-lacunar stroke (OR 3.48 vs OR 0.67 in all other studies combined). We should not discount the findings of this large population based study that contained 38% of all patients. However, we noted two problems: cognition was assessed less than 1 month after stroke and the OR of cognitive impairment in lacunar stroke changed over the study period, from 10.1 in 1991–1996 to 1.51 in 2003–2008, implying a change in methodology over time.

Our study has limitations. We lacked the resources to contact the authors to obtain original patient data on risk factors or missing values. However, it appeared that most of the missing information had not been collected.

This study's strengths include a comprehensive literature review amassing data relating to 2860 lacunar stroke patients and 4275 non-lacunar controls, including studies published in Asia, Europe and America. We did not exclude any studies because we could not obtain the papers or translate them into English.

There is no ideal way of assessing cognition after stroke. Many disabled patients will not be able to comply with detailed neuropsychological testing, yet a screening test may miss subtle cognitive decline and dementia. Other factors apart from dementia and MCI may affect cognitive assessment after stroke such as depression and fatigue; lacunar stroke is related to cerebral SVD which is associated with late life depression.^38^ An apparent association between dementia and lacunar stroke may be related to undiagnosed pre-stroke cognitive impairment or the stroke unmasking the presence of SVD. Few studies accounted for depression, premorbid IQ, pre-stroke cognitive decline, quantified WML or presented their data on cognition by WML load, so we were unable to assess whether the cognitive impairment could be explained by other factors. An apparent association between lacunar stroke and cognitive impairment may be due to survivorship bias as patients with a (often more severe) cortical stroke are less likely to survive the acute post-stroke period.

There is a lack of information on long term prognosis yet the chances of continuing cognitive decline after lacunar stroke may be higher than in non-lacunar stroke due to the differences in underlying pathology: SVD affects the brain diffusely whereas non-lacunar stroke often has an extracranial cause. The prognosis is important to patients who are cognitively impaired after lacunar stroke who wish to know if they will recover, to plan stroke services and to enable the planning of sample size and length of follow-up in future interventional studies.

Future research should focus on previously cognitively intact patients with a first lacunar stroke, extend follow-up for as long as practicable, subtype the stroke on clinical grounds aided by early MRI–DWI, account for confounding factors including differences in vascular risk factors, premorbid IQ, pre-stroke cognitive decline and depression, and validated imaging biomarkers of SVD. Analysis should calculate the odds of dementia free survival, which would reduce survivorship bias.

## Supplementary Material

Web supplement

Web supplement

Web supplement
