# Supplementary material for: Cognitive impairment after lacunar stroke: systematic review and meta-analysis of incidence, prevalence and comparison with other stroke subtypes
Source: J Neurol Neurosurg Psychiatry. 2013 Mar 1;84(8):893–900. doi: 10.1136/jnnp-2012-303645 (PMC3717603; doi:10.1136/jnnp-2012-303645)
Supplement: Web supplement [file jnnp-2012-303645-s1.pdf]

Supplementary information:

Search Strategy:

Neuropsychological Tests OR

Cognition Disorders OR

Dementia OR

Cognition OR

Dementia, Vascular OR

Dementia, Multi-Infarct OR

Mild Cognitive Impairment.mp OR

Post Stroke Dementia

AND

Brain ischemia OR

Brain infarction OR

Brain stem infarctions OR

Cerebral infarction OR

hypoxia-ischemia, brain/ or stroke/ OR
