# Supplementary figures and images for: Cognitive impairment after lacunar stroke: systematic review and meta-analysis of incidence, prevalence and comparison with other stroke subtypes
Source: J Neurol Neurosurg Psychiatry. 2013 Mar 1;84(8):893–900. doi: 10.1136/jnnp-2012-303645 (PMC3717603; doi:10.1136/jnnp-2012-303645)

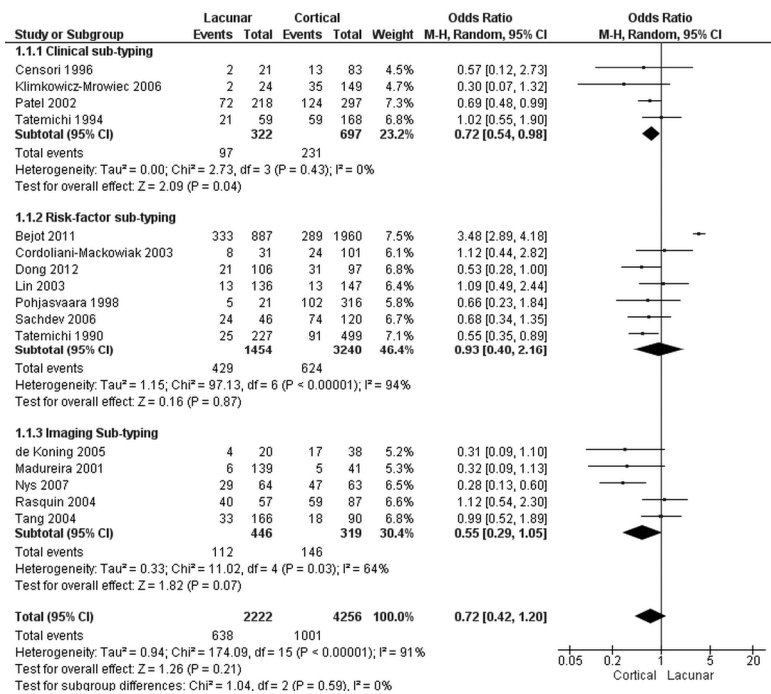

Supplement: Web supplement [file jnnp-2012-303645-s2.pdf]
