# Supplementary material for: Cognitive impairment after lacunar stroke: systematic review and meta-analysis of incidence, prevalence and comparison with other stroke subtypes
Source: J Neurol Neurosurg Psychiatry. 2013 Mar 1;84(8):893–900. doi: 10.1136/jnnp-2012-303645 (PMC3717603; doi:10.1136/jnnp-2012-303645)
Supplement: Web supplement [file jnnp-2012-303645-s3.pdf]

Supplementary Table: Excluded studies and reasons

Reason for study exclusion	***Studies excluded***
Used post-mortem pathology to sub-type the stroke	Gamaldo A, Moghekar A, Kilada S, *et al*. Effect of a clinical stroke on the risk of dementia in a prospective cohort *Neurology* 2006;**67**:1363–9.
Included haemorrhagic as well as ischaemic stroke in the lacunar group	Altieri M, Di Piero V, Pasquini M, *et al.*Vanacore N, Vicenzini E et al. Delayed poststroke dementia: A 4-year follow-up study. *Neurology* 2006; **62**:219–7
Only included a subset of stroke patients with one specific lacunar syndrome,	Grau-Olivares M, Arboix A, Junque C, *et al.*. Progressive gray matter atrophy in lacunar patients with vascular mild cognitive impairment. *Cerebrovas Dis* 2010;**30**:157–66.<br>van Zandvoort MJ, De Haan EH, Kappelle LJ. Chronic cognitive disturbances after a single supratentorial lacunar infarct. *Neuropsychiatry Neuropsychol Behav Neurol* 2001;**14**:98–102.
Only tested a specific cognitive domain that is not tested in regular clinical practice	Anderson JF, Saling MM, Donnan GA. Chronic information-processing changes in individuals with a first-ever clinical lacunar syndrome. *Cognitive & Behavioral Neurology* 2008;**21**:236–41
Excluded patients who were cognitively impaired post-stroke	Desmond DW, Moroney JT, Sano,M, *et al*. Incidence of dementia after ischemic stroke: results of a longitudinal study. *Stroke* 2002;**33**:2254–60.<br>Allan LM, Rowan EN, Firbank MJ, *et al*. Long-term incidence of dementia, predictors of mortality and pathological diagnosis in older stroke survivors. *Brain* 2011; **134**:3716–27<br>Bour A, Rasquin S, Limburg M, *et al.*. Depressive symptoms and executive functioning in stroke patients: a follow-up study. *Int J Geriatr Psychiatry* 2011;**26**:679–86.
Only recurrent stroke patients	Arboix A, Font A, Garro C, *et al.*. Recurrent lacunar infarction following a previous lacunar stroke: a clinical study of 122 patients. *J Neurol Neurosurg Psychiatry* 2009;**78**:1392–4
